# Prolonged Fever of Unknown Origin With Suspicion of Adult-Onset Still’s Disease: A Diagnostic Challenge

**DOI:** 10.14740/jmc5291

**Published:** 2026-04-29

**Authors:** Janice Tong, Jerry Yue

**Affiliations:** a Northeast Georgia Medical Center, Gainesville, GA 30501, USA

**Keywords:** Fever of unknown origin, Still’s disease, Adult-onset, Rheumatology, Fevers

## Abstract

We report a 72-year-old patient with intermittent fever for 7 months, accompanied by leukocytosis, tachycardia, myalgias and arthralgias. Prior evaluations—including imaging, cultures, bone marrow biopsy, serologies, and autoimmune panels—were unrevealing. The patient’s persistent fever was previously diagnosed as fever of unknown origin (FUO), which is defined by a core temperature exceeding 38.0 °C for more than 3 weeks with no defined causes during ≥ 3 inpatient days or ≥ 3 outpatient visits. The patient’s fever initially improved with steroids. During the hospital course, empiric antibiotics were started and discontinued following negative blood cultures, and prednisone was reinitiated at discharge. There was high suspicion for atypical adult-onset Still’s disease (AOSD), and the patient was treated in the outpatient setting with steroids and tocilizumab. This case highlights the importance of systematic evaluation, multidisciplinary collaboration, and careful selection of pharmacological therapy and invasive procedures in the workup of FUO, especially in an elderly patient. The case also illustrates the challenges in diagnosing AOSD due to the highly variable clinical presentation and low prevalence, and further outlines current treatment recommendations for AOSD, which include corticosteroid therapy in combination with an interleukin (IL)-6 inhibitor.

## Introduction

Fever of unknown origin (FUO) is a term used in medicine with nuances, and 8–30% of cases remain without clear etiology after extensive workup [1]. There is no universally accepted definition of fever; however, according to Harrison’s Principles of Internal Medicine, it is defined as a core (rectal) temperature of 37.5–38.3 °C (99.5–100.9 °F), an axillary temperature > 37.2 °C (> 99 °F), a morning oral temperature > 37.2 °C (> 99 °F), or a late afternoon oral temperature > 37.7 °C (> 99.9 °F), with lower thresholds applied in frail elderly individuals [2]. It is one of the most used vital signs in any clinical setting for patient evaluation and is an important component in many criteria guiding management such as systemic inflammatory response syndrome (SIRS) and sepsis criteria [3]. Patients presenting with fever require prompt investigation to find the source, as some underlying etiology have detrimental consequences. However, not all causes of fever are identifiable during the initial evaluation, leading to the classification known as FUO.

FUO is defined by a core temperature exceeding 38.0 °C for more than 3 weeks with no defined causes despite exhaustive investigations during ≥ 3 inpatient days or ≥ 3 outpatient visits [4]. The stringent, time-based criteria align with contemporary models of care and serve as safeguards to exclude fevers from viral or benign infectious etiology [5]. The diagnosis requires a negative initial evaluation, beginning with history and physical examination, followed by key labs including inflammatory markers, blood cultures, urinalysis, and urine cultures [4]. If these initial tests are unrevealing, more specific investigations may be pursued, including cerebrospinal fluid analysis, bone marrow biopsy, and various imaging modalities. Emerging evidence supports the earlier use of such imaging in the evaluation process [6]. When noninvasive testing fails to provide answers, tissue biopsy becomes the most useful diagnostic procedure among invasive methods. The major diagnostic categories include infections, autoimmune or autoinflammatory diseases, malignancies, miscellaneous causes, and cases that remain unexplained [7]. Retrospective data indicate that the median duration of undiagnosed FUO is approximately 24.6 weeks (about 5.7 months), with up to 75% of such cases resolving spontaneously and carrying an excellent prognosis [8].

This report highlights a patient with FUO persisting for about 7 months, for whom the cause remains unidentified despite comprehensive noninvasive and invasive evaluations conducted across multiple specialties.

## Case Report

### Investigations

A 72-year-old male patient presented to the emergency department (ED) for intermittent fever that started 7 months ago; the current episode started the day prior. The temperature at home was 38.9 °C with no relief from over-the-counter medications. The patient was tachycardic on presentation with a heart rate of 129 and febrile with an oral temperature of 39.4 °C. He had occasional myalgias of the thighs as well as a history of joint pain and stiffness. He denied rashes, nausea, vomiting, diarrhea, headache, abdominal pain, chest pain, shortness of breath, or back pain. The patient had no recent sick contacts or international travels, and no past medical history of cancer or autoimmune diseases. Lab workup was significant for a white blood cell count of 44.7 × 10^3^/µL, polymorphonuclear neutrophil count (PMN) of 95%, hemoglobin of 11.1 g/dL, platelet count of 542 × 10^3^/µL, creatinine of 1.5 mg/dL, ferritin of 434.5 ng/mL, lactate of 3.1 mmol/L, erythrocyte sedimentation rate of 71 mm/h, and C-reactive protein of 6.2 mg/dL. Liver function tests were normal. The patient was admitted for sepsis workup and initially managed with fluids and empiric antibiotics.

The patient has had multiple hospitalizations prior to the current encounter for the same complaint and clinical presentation. Labs have presented similar trends on prior admissions with, notably, ferritin as high as 1,083.4 ng/mL. Extensive prior workup had been completed with multiple specialties consulted. During prior workup, imaging revealed only significant findings of signs of inflammation of the prostate; the patient consequently underwent transurethral resection/excision of prostate with unroofing of a prostatic abscess. Postoperative imaging showed resolution of the prostate fluid collection, but fever continued to persist; therefore, prostate etiology was ruled out as the source of fever. As other infectious etiologies were suspected to be the cause of fever during previous hospitalizations, extensive infectious diagnostic testing was conducted according to the recommendation of infectious disease (ID) physicians. Initial urine cultures, blood cultures, urine drug screens, and upper respiratory viral panels were all negative. No vegetations or signs of endocarditis were seen on echocardiogram. Further specialized infectious workup results were negative, including lumbar punctures, 1,3-β-D-glucan assay, serum aspergillus galactomannan, urine histoplasma antigen, serum cryptococcus antigen, Bartonella serology, tick-borne disease antibody, Brucella serology, Q fever serology, Whipple polymerase chain reaction (PCR), mycoplasma serology, tuberculosis interferon (IFN), Chlamydia serology, rapid plasma reagin, human immunodeficiency virus (HIV) test, and Epstein-Barr virus serology. Cytomegalovirus testing was not performed. Therefore, infection was thought not to be the cause of fever, as the patient remained febrile despite completion of multiple courses of antibacterial therapies during prior hospital stays, and exhaustive diagnostic workup listed found no evidence of bacterial, viral, or fungal infection (Table 1). Hematology and oncology specialists were also consulted during prior hospitalizations to investigate potential malignancy as the cause of fever. Positron emission tomography/ computed tomography (PET/CT) did not show results consistent with malignancy. Bone marrow biopsy was negative for acute leukemia, lymphoma, and plasma cell neoplasm. Serum protein electrophoresis and urine protein electrophoresis results were not suggestive of multiple myeloma. As infection and malignancy were both excluded, rheumatology was brought on board to evaluate autoimmune and autoinflammatory causes during the patient’s evaluation at a quaternary care facility for a second opinion. From a rheumatologic workup, negative antinuclear antibody (ANA) ruled out connective tissue diseases, negative rheumatoid factor (RF) and lack of synovitis excluded rheumatoid arthritis, absence of muscle weakness and cutaneous manifestations excluded myositis, lack of temporal artery/forehead/scalp tenderness yielded low suspicion for giant cell arteritis. The consulted rheumatologist at the time also expressed low suspicion for Behcet’s, polymyalgia rheumatica, crystalline arthropathies, hemophagocytic lymphohistiocytosis, or macrophage activation syndrome due to the absence of classic symptoms associated with the listed diseases, such as ulcers, uveitis, vasculitis, joint inflammation, hepatosplenomegaly, and hematologic dysfunction respectively. The patient was consequently recommended to consider enrollment in a National Institutes of Health (NIH) study on undiagnosed periodic fever syndromes, which he did not pursue [9]. The patient was not started on systemic steroids or immunosuppression at the quaternary center due to low suspicion for autoimmune processes at the time. A steroid trial was initiated two hospitalizations later, as fever persisted despite antibiotics and no clear underlying etiology. Fever was observed to improve during the steroid trial; therefore, the patient was discharged 9 days prior to the current presentation on a prednisone taper course, including prednisone 40 mg daily for 4 days and 20 mg daily for 7 days (Table 1).

**Table 1:** All Pharmacological Treatments the Patient Received Since the Initial ED Presentation, Including Name of Treatment, Dose, Route, Frequency, and Duration of the Course

Time from initial presentation	Pharmaceutical treatment	Dose/route/frequency	Course
About 1 month	Ertapenem	1,000 mg IV every 24 h	7 days
	Piperacillin/tazobactam	4.5 g in NaCl 0.9% 100 mL IVPB	Once
	Cefepime	2,000 mg/50 mL IVPB	Once
	Vancomycin	1,000 mg IV every 12 h	5 days
	Ciprofloxacin	400 mg IV every 12 h	3 days
	Ampicillin	2,000 mg IV every 6 h	3 days
About 2 months	Vancomycin	1,500 mg IV every 18 h	5 days
	Daptomycin	8 mg/kg IV every 24 h	11 days
	Vancomycin	1,500 mg IV every 18 h	2 days
		1,000 mg IV every 12 h	18 days
	Azithromycin	500 mg oral daily	3 days
	Levofloxacin	500 mg oral daily	13 days
About 3 months	Meropenem	1,000 mg IV every 8 h	11 days
	Doxycycline	100 mg oral twice daily	20 days
	Levofloxacin	500 mg oral daily	15 days
About 4 months	Vancomycin	1,000 mg IV every 12 h	3 days
	Ceftriaxone	2,000 mg IV every 24 h	3 days
About 5 months	Ceftriaxone	2,000 mg IV every 24 h	3 days
	Prednisone	40 mg oral daily	4 days
		20 mg oral daily	7 days
About 6 months	Piperacillin/tazobactam	4.5 g in NaCl 0.9% 100 mL IVPB	Once
	Vancomycin	1,500 mg IV every 24 h	4 days
	Cefepime	2,000 mg IV every 8 h	4 days
	Prednisone	30–40 mg oral daily	Ongoing
About 7 months	Tocilizumab	4 mg/kg IV every 4 weeks	Ongoing

ED: emergency department; IV: intravenous; IVPB: intravenous piggyback.

During the current admission, body temperature was monitored orally, as during admission. Consistent with prior hospitalizations, the current ID physician believed the presenting picture was not suggestive of infectious etiology given negative blood cultures and unremarkable imaging. Drug-induced fever was considered within the differential diagnosis but was ruled out due to negative urine drug screens and the patient not being on any home medications known to cause fever. Rheumatology and oncology consultations were again recommended. Upon evaluation of the patient and review of prior medical records, the rheumatology team believed that the fever was highly likely secondary to underlying autoimmune or autoinflammatory conditions. Differential diagnoses included giant cell arteritis, adult-onset Still’s disease (AOSD), and autoinflammatory diseases. A temporal artery biopsy was performed to investigate giant cell arteritis; the biopsy was negative for arteritis and giant cells, thus ruling out the presumed number one differential diagnosis on the list.

### Treatment

Antibiotics were discontinued following no growth from blood culture at 48 h, per ID recommendation. Steroid therapy was reinitiated, using prednisone 40 mg daily, which improved fever and leukocytosis. All treatments are listed in detail in Table 1.

### Follow-up and outcomes

**Figure 1:**
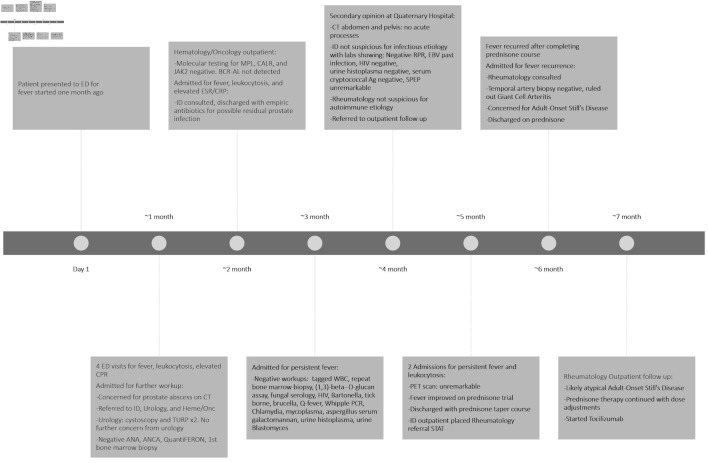
Timeline demonstrating all ED visits, admissions, laboratory/imaging diagnostic tests, and consultations from the first ED presentation to the most recent outpatient follow-up. ED: emergency department; ESR: erythrocyte sedimentation rate; CRP: C-reactive protein; ID: infectious disease; CT: computed tomography; RPR: rapid plasma reagin; EBV: Epstein–Barr virus; HIV: human immunodeficiency virus; SPEP: serum protein electrophoresis; TURP: transurethral resection of the prostate; ANA: antinuclear antibody; ANCA: anti-neutrophil cytoplasmic antibody; WBC: white blood cell; PCR: polymerase chain reaction; PET: positron emission tomography.

As fever and leukocytosis resolved and the patient remained stable, emergent further workup was not warranted. Despite the involvement of multiple experienced physicians, no clear final diagnostic consensus was reached. The patient was once again discharged with a prednisone taper course and a referral to rheumatology for further outpatient workup. During outpatient rheumatology follow-ups, the patient was suspected to have underlying atypical AOSD. The rheumatologist noted that, although the patient does not have other typical features of this condition like rashes, hepatosplenomegaly, and elevated liver function tests, the markedly elevated inflammatory markers (erythrocyte sediment rate and C-reactive protein level), elevated white blood cell count, and the fever pattern were indeed more consistent with AOSD. The rheumatologist adjusted the patient’s prednisone course and started the patient on a tocilizumab infusion, an interleukin (IL)-6 receptor antagonist, as a steroid sparing treatment, to avoid adverse effects of long-term steroid, including increased risk of infection, insomnia, and weight gain. The timeline from the first ED encounter to the most recent outpatient follow-up visit is depicted in Figure 1.

## Discussion

The presented case is an example of FUO workup in an elderly patient, which requires special considerations due to a different spectrum of underlying conditions and lower tolerances for exhaustive FUO workup. For elderly patients, FUO workup is recommended to start with a targeted history and physical examination focusing on common causes of FUO in elderly patients, including intra-abdominal diseases, cardiac disorders, tuberculosis, musculoskeletal disorders, and cancers. FUO in the elderly may be considered with body temperatures less than 38.3 °C, or at least within the range of 37.5–38.3 °C, due to blunted fever responses with age and serious infections. The initial noninvasive workup includes chest radiographs, basic laboratory studies, imaging, blood cultures, and echocardiography. More specific, invasive diagnostic studies should be performed based on clinical suspicion from initial tests [10].

Differentials considered throughout this patient’s 7 months of multiple hospitalizations included infectious causes, drug-induced fever, neoplastic causes, and inflammatory causes. With negative routine and specialized infectious testing, lack of antibiotic response, negative urine drug screens, review of home medications with no known adverse fever effects, and negative hematologic workup thus far, infectious, drug-induced, and neoplastic causes have been effectively ruled out. The repeated resolution of fever with the use of steroids suggests that autoimmune or autoinflammatory diseases are the most probable cause of FUO in this patient. This reflects the shift from infectious disease as the predominant cause of FUO to autoimmune or autoinflammatory conditions observed in recent years [7]. Autoimmune and autoinflammatory conditions considered included Behcet’s, polymyalgia rheumatica, crystalline arthropathies, hemophagocytic lymphohistiocytosis, macrophage activation syndrome, giant cell arteritis, and AOSD. Given the patient’s clinical presentation, absence of classic symptoms, and negative temporal artery biopsy, AOSD was the leading suspected etiology of the patient’s fever.

A retrospective cohort study conducted in China, with a median patient age of 38.7 years, showed that AOSD is the most frequent noninfectious inflammatory cause of FUO, accounting for over half of connective tissue disease cases in some series [11]. AOSD is a rare systemic autoinflammatory disorder with an incompletely understood pathogenesis, with an estimated prevalence of 0.2–0.4 cases per 100,000 people [12, 13]. The majority of cases occur in females and between 16 and 35 years of age, although approximately 10% present after age 50 [14]. The diagnosis of AOSD is typically made after exclusion of infections, malignancies, and other autoimmune conditions [15]. The disease is characterized by high spiking fever, sore throat, evanescent skin rash, arthritis, neutrophilic leukocytosis, markedly elevated ferritin level, negative ANA and RF, and abnormal liver function tests. However, not all patients experience all the listed symptoms; fever occurs in 60–90% of patients, joint pain in 70–100%, skin rash in 60–90%, and sore throat in 70% [16]. Other symptoms, such as pharyngitis, odynophagia, lymphadenopathy, splenomegaly, myalgia, pleuritis, or abdominal pain, vary among the patients. Clinical manifestations of AOSD are highly variable, making diagnosis difficult. The two widely used diagnostic criteria are the Yamaguchi and Fautrel classification criteria [12]. The Yamaguchi criteria for AOSD include major criteria consisting of intermittent fever of at least 39 °C lasting 1 week or longer, arthralgias or arthritis lasting 2 weeks or longer, rash, and leukocytosis (≥ 10 × 10^3^/µL with ≥ 80% PMNs). Minor criteria consist of sore throat, recent development of lymphadenopathy, hepatomegaly or splenomegaly, abnormal liver function, and negative tests for ANA and RF. Diagnosis of AOSD requires five or more criteria, of which two must be major [17]. Major criteria within Fautrel classification criteria consist of spiking fever ≥ 39.0 °C, arthralgias, transient erythematous rash, sore throat, PMN ≥ 80%, and glycosylated ferritin ≤ 20%. Minor criteria consist of maculopapular rash and leukocytosis (≥ 10 × 10^3^/µL). Exclusion criteria consist of infections, malignancies, and other rheumatic diseases. Diagnosis of AOSD requires at least four major criteria or three major and two minor criteria [18]. The Yamaguchi criteria has been used in 98.5% of AOSD studies [19]. It has been shown to have higher sensitivity, specificity, positive predictive value, and negative predictive value than the Fautrel criteria [15].

Although not quite meeting either diagnosis criteria, the patient in this case experienced most of the characteristics listed in the two criteria, including fever ≥ 39.0 °C, arthritis, leukocytosis ≥ 10 × 10^3^/µL, PMN ≥ 80%, and negative ANA and RF [17, 18]. Furthermore, ferritin, which has a role in cytokine production, is characteristically elevated in AOSD, typically up to 4–5 times the upper limits of normal [20]. Though not as high, the patient’s ferritin level was markedly elevated at 1,083.4 ng/mL on previous admissions. Glycosylated ferritin percentage, which is seen in the Fautrel classification criteria, was not tested in this patient for reasons not documented by the treating rheumatologist. Partial fulfillment of the classification criteria raised strong suspicion for atypical AOSD in this patient. This non-classic presentation is consistent with reports of AOSD in individuals over 70 years, in whom disease manifestations often deviate from conventional criteria, contributing to a higher incidence of delayed diagnosis [14].

The Yamaguchi and Fautrel classification criteria establish the diagnosis of Still’s disease based on clinical features. More recent studies have identified several inflammatory mediators as potential diagnostic biomarkers, with IL-18 and S100 proteins among the most extensively investigated [21]. Although these assays are utilized in select tertiary centers, the absence of standardized and validated thresholds limits their widespread adoption, as was the case here [22].

Although the pathogenesis of AOSD remains incompletely elucidated, immune effector cells—including neutrophils, macrophages, and T cells—together with pro-inflammatory cytokines, particularly IL-1, IL-6, IL-18, and IFN-γ, are central to disease development. Glucocorticoids remain the first-line therapy; however, the introduction of molecularly targeted agents has transformed management. IL-1 and IL-6 inhibitors have demonstrated efficacy in controlling disease activity and minimizing glucocorticoid exposure [23]. The use of tocilizumab, an IL-6 receptor antagonist, in the patient of this case is consistent with recommendations from the European Alliance of Associations for Rheumatology and the Pediatric Rheumatology European Society joint task force, which advocates for early initiation of IL-targeted therapy (IL-1 or IL-6 inhibitor) in combination with a short course of glucocorticoids as the optimal management strategy for Still’s disease [21]. In refractory cases, emerging strategies such as IL-18 binding protein, Janus kinase (JAK) inhibitors, and IFN-γ blockade may be considered [24].

In analyzing the management of this patient, the use of multiple courses of empiric antibiotics for this patient potentially deviates from the current recommendation of reserving its use for patients with neutropenia or who are severely immunocompromised or rapidly deteriorating. Empiric antibiotic use in FUO should be carefully considered, as it can predispose patients to antimicrobial resistance, delay diagnosis, and cause false reassurance if fever is in-fact benign. In cases where a certain diagnosis is highly suggested by initial evaluation, clinicians should use clinical judgment to decide the need for drug challenge. For elderly patients deteriorating quickly, 10–14 days of broad-spectrum antibiotics are generally recommended following diagnostic workup [10]. Another point of contention in this case is whether temporal artery biopsy was truly indicated, as patient did not endorse headache or jaw claudication. This decision is in favor of an evidence-based review that proposed temporal-artery biopsies in elderly patients with unresolved FUO to look for temporal arteritis and may be considered on a case-by-case basis, as the disease has serious ramifications [25]. Generally, elderly patients with FUO who are older than 60, with anemia, a hallmark elevated erythrocyte sedimentation rate (> 40 mm/h), and elevated alkaline phosphatase levels have a high pretest probability of temporal arteritis and warrant consideration of a temporal artery biopsy. However, temporal artery biopsies are only positive in 60–80% of patients due to possible skip lesions [10]. Lastly, alternative medication management could have been pursued for this patient if drug fever was suspected. Drug fever was ruled out in this patient through urine drug screens and review of home medications. Management of FUO in patients recommends that nonessential drugs be immediately discontinued, along with essential drugs, if the fever continues to persist. Persistence of fever 72 h after drug discontinuation would effectively rule out drug-induced fever [10].

### Conclusions

The patient presented with a 7-month history of intermittent fever, myalgias, and arthralgias. This case exemplifies FUO, a clinical entity that necessitates a structured, stepwise diagnostic approach integrating detailed patient history, targeted clinical evaluation, and multidisciplinary collaboration. In this instance, coordinated involvement of multiple specialties helped carefully appraise and appropriately select invasive investigations, avoid unnecessary prolonged antibiotic therapy, and facilitate the identification of an autoimmune or autoinflammatory etiology. With partial fulfillment of the Yamaguchi and Fautrel criteria, atypical AOSD is the leading suspected etiology, highlighting the importance of systematic evaluation of FUO, especially in elderly patients, and consideration of autoinflammatory syndromes in prolonged unexplained fever.

## Data Availability

The data supporting the findings of this study are available from the corresponding author upon reasonable request.

## References

[1] Mourad O, Palda V, Detsky AS (2003). A comprehensive evidence-based approach to fever of unknown origin. Arch Intern Med.

[2] Mackowiak PA, Chervenak FA, Grunebaum A (2021). Defining fever. Open Forum Infect Dis.

[3] Singer M, Deutschman CS, Seymour CW, Shankar-Hari M, Annane D, Bauer M, Bellomo R (2016). The third international consensus definitions for sepsis and septic shock (Sepsis-3). JAMA.

[4] Knockaert DC, Vanderschueren S, Blockmans D (2003). Fever of unknown origin in adults: 40 years on. J Intern Med.

[5] Haidar G, Singh N (2022). Fever of unknown origin. N Engl J Med.

[6] Singh SB, Shrestha N, Bhandari S, Shrestha S, Shrestha B, Shrestha N, Rijal S (2024). [(18)F]FDG PET/CT for identifying the causes of fever of unknown origin (FUO). Am J Nucl Med Mol Imaging.

[7] Antoniadou C, Gavriilidis E, Chatzopoulos P, Gkouliavera M, Skendros P (2025). Fever and inflammation of unknown origin in the 21st century. Eur J Intern Med.

[8] Tan Y, Liu X, Shi X (2019). Clinical features and outcomes of patients with fever of unknown origin: a retrospective study. BMC Infect Dis.

[9] https://clinicaltrials.gov/study/NCT00001373.

[10] Tal S, Guller V, Gurevich A (2007). Fever of unknown origin in older adults. Clin Geriatr Med.

[11] Zhai YZ, Chen X, Liu X, Zhang ZQ, Xiao HJ, Liu G (2018). Clinical analysis of 215 consecutive cases with fever of unknown origin: a cohort study. Medicine (Baltimore).

[12] Macovei LA, Burlui A, Bratoiu I, Rezus C, Cardoneanu A, Richter P, Szalontay A (2022). Adult-onset Still's disease-a complex disease, a challenging treatment. Int J Mol Sci.

[13] Shen Y, Jia J, Teng J, Yang C, Hu Q (2025). Advancing personalised precision treatment for still's disease based on molecular characteristics and disease progression. Lancet Rheumatol.

[14] Mollaeian A, Chen J, Chan NN, Nizialek GA, Haas CJ (2021). Adult onset Still's disease in the elderly: a case-based literature review. BMC Rheumatol.

[15] Tomaras S, Goetzke CC, Kallinich T, Feist E (2021). Adult-onset still's disease: clinical aspects and therapeutic approach. J Clin Med.

[16] Gerfaud-Valentin M, Jamilloux Y, Iwaz J, Seve P (2014). Adult-onset Still's disease. Autoimmun Rev.

[17] Yamaguchi M, Ohta A, Tsunematsu T, Kasukawa R, Mizushima Y, Kashiwagi H, Kashiwazaki S (1992). Preliminary criteria for classification of adult Still's disease. J Rheumatol.

[18] Fautrel B, Zing E, Golmard JL, Le Moel G, Bissery A, Rioux C, Rozenberg S (2002). Proposal for a new set of classification criteria for adult-onset still disease. Medicine (Baltimore).

[19] Balay-Dustrude E, Marques MC, Appenzeller S, Bracaglia C, Dedeoglu F, Eloseily E, Jimenez PM (2026). Clinical features and outcome measures across still disease (Systemic Juvenile Idiopathic Arthritis and Adult-Onset Still Disease) cohorts worldwide: a systematic literature review. J Rheumatol.

[20] Mehta B, Efthimiou P (2012). Ferritin in adult-onset still's disease: just a useful innocent bystander?. Int J Inflam.

[21] https://acrabstracts.org/abstract/clinical-and-biological-characteristics-of-children-and-adults-affected-with-stills-disease-a-systematic-review-and-meta-analysis-informing-the-2023-eular-pres-recommendations-for-the-diagno/.

[22] Fautrel B, Mitrovic S, De Matteis A, Bindoli S, Anton J, Belot A, Bracaglia C (2024). EULAR/PReS recommendations for the diagnosis and management of Still's disease, comprising systemic juvenile idiopathic arthritis and adult-onset Still's disease. Ann Rheum Dis.

[23] Suzuki K, Kaneko Y (2025). Updates on the pathogenesis and molecular-targeted therapies of Still's disease. Immunol Med.

[24] Pietsch D, Savic S (2026). Still's disease and autoinflammation: positioning an inflammatory syndrome on the autoinflammation-autoimmunity spectrum. Curr Rheumatol Rep.

[25] Hayakawa K, Ramasamy B, Chandrasekar PH (2012). Fever of unknown origin: an evidence-based review. Am J Med Sci.

